# Observational Gait Assessment Scales in Patients with Walking Disorders: Systematic Review

**DOI:** 10.1155/2019/2085039

**Published:** 2019-10-31

**Authors:** Carmen Ridao-Fernández, Elena Pinero-Pinto, Gema Chamorro-Moriana

**Affiliations:** Department of Physiotherapy, Research Group “Area of Physiotherapy CTS-305”, University of Seville, 41009 Seville, Spain

## Abstract

**Objective:**

To compile and analyze the characteristics and methodological quality of observational gait assessment scales validated to date.

**Methods:**

*PubMed*, *Scopus*, *the Cochrane Library*, *Physiotherapy Evidence Database*, *Web of Science*, *Cumulative Index to Nursing* and *Allied Health Literature*, *Dialnet*, *Spanish Medical Index*, and *Nursing, Physiotherapy*, and *Podiatry* databases were searched up to August 2019. The main inclusion criteria were validated tools based on a conceptual framework developed to evaluate gait, validation design studies of observational scales in their entirety, and articles written in English or Spanish. Evaluators extracted descriptive information of the scales and the metric properties of the studies, which were further analyzed with *Quality Assessment of Diagnostic Accuracy Studies* (*QUADAS-2*) and *COnsensus-based Standards for the selection of health Measurement Instruments* (*COSMIN checklist*).

**Results:**

Eighteen articles based on 14 scales were included. The populations were neurological patients (72.22%), musculoskeletal disorders (11.11%), and other areas such as vestibular disorders (11.11%). The most addressed items were orthopedic aids (64.29%); phases of the gait cycle and kinematics of the leg and trunk (57.14% each one); and spatial and temporal parameters (50%). All studies analyzed criterion validity, and five included content or structural validity (27.78%). Fifteen articles considered reliability (83.33%). Regarding the seven-item scale QUADAS-2, five studies obtained six results on “low” risk of bias or “low” concerns regarding applicability. Nine articles obtained at least a “fair” result on COSMIN checklist.

**Conclusions:**

A necessary compilation of the observational gait assessment scales validated to date was conducted. Besides, their characteristics and methodological quality were analyzed. Most scales were applied in neurological signs. The most approached topics were orthopedic aids, phases of the gait cycle, and kinematics of the leg and trunk. The scale that demonstrated a higher methodological quality was *Visual Gait Assessment Scale*, followed by CHAGS, *Salford Gait Tool*, and *Edinburgh Visual Gait Score*.

## 1. Introduction

Assessment methods of mobility are necessary to identify structural, biomechanical, and functional limitations, develop treatment plans, and assess the effect of treatments [1, 2]. This is why numerous efficient, valid, and reliable evaluation procedures have been designed [3]. Some of them concentrate on the characteristics of the gait pattern [4, 5]. Gait recovery is often directed by physiotherapists and constitutes an important part of trauma patients' treatment such as subjects who undergo knee or hip surgery and neurological disorders, such as patients who have had a stroke [3, 6]. To carry out this recovery process, physiotherapists need to examine, objectify, and document their patients' gait [7].

This need to functionally assess gait has given rise to the creation of sophisticated assessment mechanisms [4, 8]. Some of the most common methods are electromyography, which registers the electrical activity of the muscle [9], and 3D systems of motion analysis, which allows reconstruction of the position and orientation of corporal segments in the space [10]. The instrumented gait analysis has been accepted as the Gold Standard for the evaluation of this function, as it provides reliable and accurate information in the three planes movement [3, 11]. However, these new techniques have disadvantages that distance them from the clinical setting [4]. Besides having a high economical cost and difficult access as they are not available for all professionals, the instrumented analysis is complex, requires time resources, and requires high level of skill in its use [3, 4, 12, 13].

The disadvantages of instrumented gait analysis have led to the development of a variety of observational tools [14]. Visual gait assessment by scales is more viable than instrumented analysis in the clinical setting since it has low cost and does not require specialized equipment or location [11, 13, 15]. It is an available and interesting help, specifically when standardised scales are used, as well as an accurate and reliable tool that issues clinical assessments [16]. These advantages have led to the creation of numerous visual gait evaluation scales aimed at patients with neurological disorders such as Parkinson's disease [4] or cerebral palsy (CP) [12], in elderly subjects or patients with some orthopedic disorders [17]. All scales aim for a complete and objective evaluation of gait despite having different assessment forms and content. Some of them evaluate falls and balance, which could be relevant for the analysis of patients with Parkinson's disease or vestibular disorders [4]. Other scales focus on the study of kinematics or the gait cycle. There are also assessment forms that analyze gait parameters as arm swing and fluency and take into account the use of assistive devices, frequently employed during gait recovery [14]. Observational gait assessment scales have become an effective clinical alternative due to its speed and ease of use, probably becoming the most commonly used method [3].

The widespread use of these assessment measures highlights the need for developing and validating scales as well as integration studies, data collection, analysis, and their dissemination [16]. Therefore, and according to authors such as Toro [14], it can be said that there is a lack of documentation that objectively collects scientific information about visual scales, which are frequently difficult to access in a clinical setting. However, we have found some review studies published in recent years. Some of these papers focus on the analysis of a single assessment scale, as in the case of the work published by Bartels et al. [18] in 2013, about the six-minute walk test. Other researchers analyzed the existing scientific evidence in evaluating the progress of a particular patient population. Among them are the publications by Ferrarello et al. [19], conducted in 2013, Hawkins and Riddick [20], conducted in 2018, and Rathinam et al. [21], conducted in 2014. However, we found no systematic reviews on unrestricted observational gait tools regarding the pathology or study population. For this reason, the aim of this systematic review was to compile observational gait assessment scales validated to date and to analyze their characteristics and methodological quality.

## 2. Materials and Methods

The method was based on the PRISMA protocol [22].

### 2.1. Search Strategy

An electronic search of *PubMed*, *Scopus*, *the Cochrane Library*, *Physiotherapy Evidence Database* (*PEDro*), *Web of Science* (*WOS*) *Cumulative Index to Nursing and Allied Health Literature* (*CINAHL*), *Dialnet*, *Spanish Medical Index* (*IME*) and *Nursing*, and *Physiotherapy* and *Podiatry* (*ENFISPO*) was performed up to August 2019.

All the key words used in this study are included in Mesh (Medical Subject Headings) for English language or DeCS (*Descriptores en Ciencias de la Salud*) for Spanish, except *deambulation* and *assess*. However, the authors decided to include them in our search terms as they are considered relevant in publications that dealt with the design and validation of observational analysis scales of human gait.

Table 1 show the terms applied. The full search strategies are reported in “Search strategies” in Supplementary Materials ([Supplementary-material supplementary-material-1]).

### 2.2. Study Selection

The included papers had to meet the following inclusion criteria: (1) validated tools based on a conceptual framework developed to evaluate gait, including assisted gait; (2) validation design studies of observational scales in their entirety, i.e., that did not validate isolated items; and (3) articles written in English or Spanish. Exclusion criteria were (1) those articles whose assessment scale was not available, (4) scales that did not assess gait parameters, and (3) cross-cultural adaptations.

The reviewers screened the titles and abstracts of the search results to check if studies met the preestablished inclusion criteria. The full text was acquired from those studies that met the criteria, and the exclusions were documented.

### 2.3. Data Extraction

Data extraction was carried out by one reviewer (CR) and verified by a second reviewer (GC). A table designed to detail information about observational gait assessment scales was employed, which considered author, year, country of origin, indications, operating instructions, and recommendations, study sample (employed for validation), and metric properties. Disagreements between reviewers were resolved by a third reviewer (EP), who assessed the information independently to resolve the discrepancies. Publication date, authors, and journal of publications were not blinded to the reviewers.

Afterwards, a second table included detailed information on scale items: global analysis, kinematics (arm, leg, and trunk), kinetics, spatial and temporal parameters (e.g., step length, step width, and step period), phases (of the gait cycle and the plantar support), fluency, arm swing, facing forward, center of mass/base of support displacement, gait optimization parameters (jumps, displacements, etc.), falls, balance, gait with obstacles, orthopedic aids, functional tests, footprints, pain, quality of life, psychological aspects (e.g., confidence), and care level.

### 2.4. Quality Appraisal

Two assessment scales were used to evaluate the quality of the identified scales and the methodological quality of the articles validating the tools: the scales *Quality Assessment of Diagnostic Accuracy Studies* [23] (*QUADAS-2*) and *COnsensus*-*based Standards for the selection of health Measurement Instruments* [24] (*COSMIN checklist*).

The QUADAS-2 tool, an analysis scale of diagnostic criteria validation studies, was used to assess risk of bias and applicability. The questions that make up the seven items of this scale allow us to determine whether there is a “low,” “high,” or “unclear” risk that there has been a bias in each domain or concern regarding applicability. *QUADAS-2* has been recommended by the *Agency for Healthcare Research* and *Quality*, *the Cochrane Collaboration*, and *the UK National Institute for Health and Clinical Excellence* for its use in this type of systematic reviews [23].

The *COSMIN checklist* with a 4-point scale, which classifies each assessment in a range of four levels (“excellent,” “good,” “fair,” and “poor”), was used to assess the metric properties of the studies. This scale consists of nine sections, which correspond to nine metric properties, each of which contains five to 18 items concerning aspects of design and statistical methods [24]. COSMIN checklist does not take into account the metric properties that have not been developed in papers.

## 3. Results

The literature search identified 2259 records. Most of them were found in PubMed, and the rest in Scopus, Cochrane, PEDro, WOS, CINAHL, Dialnet, IME, and ENFISPO. Following the removal of duplicates, 1753 studies were screened by title, abstract, and full text following the selection criteria. After the screening, 18 papers related to 14 observational gait scales were included in this review.

**Figure 1:**
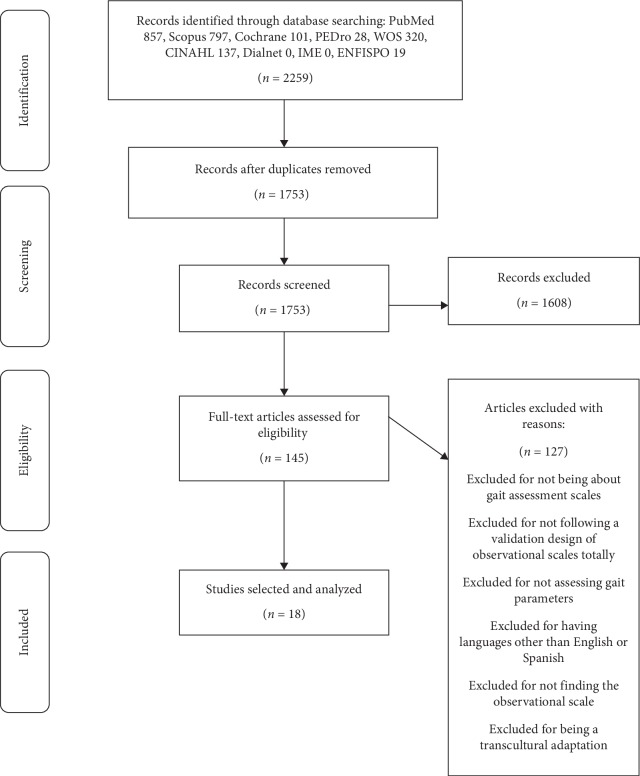
PRISMA flow diagram of the literature search results conducted up to August 2019.

Figure 1 presents the flow diagram of the study selection process. The PRISMA checklist is attached in “Prisma Statement Checklist” of Supplementary Materials ([Supplementary-material supplementary-material-1]).

It was not found any additional paper to the search strategy that accomplished the selection criteria.

### 3.1. Characteristics of the Included Studies

A summary of the descriptive data from the selected papers (author, year, country of origin, indications, operating instructions and recommendations, study sample, and metric properties) is shown in Table 2.  Table 3 contains the items located by the reviewers in the selected assessment tools.

As for the study population, 13 of the 18 articles included (72.22%) dealt with the assessment of patients with neurological signs (CP, stroke, multiple sclerosis, Parkinson's disease, hemiplegia, traumatic brain, and spinal cord injuries) [4, 12, 14, 15, 25–29, 31–36]. Two studies were found in musculoskeletal disorders (fractures of the tibial shaft and sprained ankles) (11.11%) [17, 37] and one study was about each of the following populations: vestibular disorders [34] and older adults [35] (5.56% each one).

In summary, the most addressed areas in the gait scales are, in descending order: orthopedic aids [4, 17, 25–27, 30, 33, 34, 37] (64.29%); phases of the gait cycle [4, 12, 14, 15, 25–28, 31] and kinematics of the leg [12, 14, 15, 25–27, 29, 31, 32] and trunk [4, 14, 15, 25, 26, 29–32] (57.14% each one); spatial [4, 27, 30–33, 37] and temporal [4, 17, 31–34, 37] parameters (50% of them); arm swing [4, 26, 30, 37], center of gravity (base of support displacement) [26, 31, 32, 37], and gait optimization parameters (e.g., jumps and displacements) [4, 26, 30, 34] (28.57% each one); kinematics of the arm [25, 26, 31], fluency [4, 34, 37], and gait with obstacles [4, 30, 34] (21.43% each one); balance [4, 34] and functional tests [4, 33] (14.29% each one); kinetics [32], facing forward [37], falls [4], and psychological aspects [4] (e.g., confidence); and care level [4] (7.14% each one).

Regarding the metric properties, all studies analyzed criterion validity [4, 12, 14, 15, 17, 25–37]. Five articles also considered content validity or structural validity (27.78%) [25, 26, 34, 36, 37]. Fifteen of the 18 articles included intrarater and interrater reliability or both [4, 12, 15, 17, 25–31, 33, 34, 36, 37] (83.33%). Responsiveness and internal consistency were assessed in four (22.22%) [25, 26, 29, 33] and three (16.67%) [30, 34, 37] articles, respectively.

### 3.2. Quality Appraisal

The results of QUADAS-2 tool are shown in Table 4. Of the 126 sections of *QUADAS-2* in this study (seven items for 18 papers), 86 obtained a “low” risk of bias or “low” concerns regarding applicability, 34 resulted “unclear,” and six had “high” risk of bias or “high” concerns regarding applicability. As for the included studies, five of them obtained six results on “low” risk of bias or “low” concerns regarding applicability [14, 15, 28, 29, 34]. Six papers obtained up to five of these results [4, 17, 26, 27, 36, 37]. Another five studies accumulated four [30–32, 33, 35], and the remaining two studies obtained three “low” risk of bias or “low” concerns regarding applicability [12, 25].

Table 5 contains the methodological assessment quality based on *COSMIN checklist* [24] (see Section 2.4). The studies rated “poor” for most of the sections on the scale. Nine of the 18 articles that make up this systematic review obtained at least a different result, which in all cases was “fair” [14, 17, 28, 30, 32, 34–37]. There were no cases where the results of *COSMIN checklist* were “good” and “excellent.”

Three sections were suppressed (measurement error, hypotheses testing, and cross-cultural validity) as they were not considered in any of the 18 articles included.

## 4. Discussion

This systematic review compiled functional gait assessment scales, with no time limitation, which had previously been validated. Consequently, some validation studies were performed many years ago without the scientific requirements demanded by the prestigious journals nowadays, that is, completed validations that include reliability and content, criteria (or concurrent) and construct validities, and metric properties whose calculations have been considered in this revision. Besides, the analysis performed has taken into account the methodological quality, as will be developed below.

Regarding the study population, the findings sustained that most scales were applied in neurological pathologies as CP, stroke, multiple sclerosis, Parkinson's disease, hemiplegia, and traumatic brain and spinal cord injuries. Only two assessment tools were used to analyze the gait of patients with musculoskeletal disorders, in particular, tibial fractures [17] and sprained ankles [37]. Although the latter was validated in subjects with sprained ankles, CHAGS was the only scale intended for the evaluation of aided gait with forearm crutches to partially relieve an affected member due to a musculoskeletal injury [37]. As can be observed, researchers have dwelt on the analysis of gait on neurological pathologies but not on musculoskeletal injuries [3].

As for the items included in the scales, the most used item was orthopedic aids This was approached in 9 of the 14 scales (64.29%) [4, 17, 25–27, 30, 33, 34, 37]. Moreover, one of the scales was specifically designed for the analysis of assisted gait, as mentioned above. However, it evaluated gait with forearm crutches and did not consider any other device applicable to the older and neurological population such as walkers or axillary crutches. Most of the scales considered the use of assisted devices as an isolated item where the evaluator has to indicate the aid system employed. Instead, OGS [27], GABS [4], and SCI-FAI [33] also included the independence of the subject for their displacement, by employing devices such as crutches or walkers.

Kinematics of the leg (e.g., knee progression angle and peak extension of pelvis during stance) and the trunk (e.g., peak sagittal position and trunk side flexed in swing phase) appeared in 8 of the scales (57.14%) [4, 12, 14, 15, 25–27, 29–32]. However, kinetics only appeared in one scale (7.14%) [32]. Although both kinematics and kinetics are relevant in gait analysis, kinematics is more feasible in observational evaluations [38]. The study of kinetics requires technological systems, such as force platforms or electromyography sensors, which hinder its analysis in the clinical practice [39]. Kinematics, however, allows a technological evaluation of gait as well as observational. Kinematics of the arms was the least analyzed (21.43%) [25, 26, 31] in spite of having a relevant influence in gait as a global movement. Only one of the scales that analyzed it included arm swing [26], which was contained in the 28.57% of the tools [4, 26, 30, 37].

Spatiotemporal parameters, such as step length, step width, step period, and velocity, are considered essential for gait evaluation and are useful in the functional analysis of the patient [40]. Their presence in gait scales was expected since their analysis allows correction and prevention of gait alterations. However, they were included only in half of the scales [4, 17, 27, 30, 31, 32, 33, 34, 37]. Only five scales (35.71%) considered both spatial and temporal step parameters [4, 31, 32, 33, 37]. Three of these tools were found to analyze symmetry [4, 31, 37]. The authors highlight the scale HGAF [31], which studied symmetry from the spatial and temporal point of view, with the evaluation of the symmetry of step length and step period. Its evaluation is considered relevant as a symmetrical gait is necessary for the development of a biomechanically correct gait pattern [41].

In spite of being feasible and valid for clinical practice, only GABS [4] and SCI-FAI [33] scales included functional tests (stand-walk-sit time and Romberg test and 2-minute walk test, respectively). Health professionals frequently employ these tests, together with the evaluation of step parameters, gait kinematics, gait cycle, and the rest of items that compose functional scales. These professionals have the skills to functionally evaluate gait, especially physiotherapists that are experienced in gait reeducation.

The development of a correct gait pattern requires a good dynamic balance [42]. Balance was found in two scales (GABS [4] and FGA [34]). They were designed for the analysis of subjects who suffered Parkinson's disease and vestibular disorders, respectively. In both cases, balance alteration is considered a principal sign present in patients, but it is not exclusive to them. Balance can be expected to alter in patients with lower levels of confidence. In these cases, psychological aspects can lead to a gait pattern characterized by small step length and step period, with a tendency to lose balance and fall. These three related aspects are appropriately included in the GABS scale [4].

In relation to the methodological evaluation with the *COSMIN checklist*, most of the results were rated “poor” and in no case were the results “good” and “excellent” given. These data indicate that the methodological quality of the studies has limitations. Regarding systematic reviews of observational gait analysis scales, we found only one study that used the *COSMIN checklist* in its methodology. This is the article published by Ferrarello et al. [19] in 2013, in which assessments are limited to “poor” and “fair,” the same as in our study. Unlike observational gait analysis scales, higher scores (“good” and “excellent”) are obtained in other systematic reviews, such as the studies by Bartels et al. [18] and Paiva et al. [43]. The similarity that occurs between our results and those obtained by Ferrarello [19] highlights the lack of scientific evidence that exists in visual gait assessment instruments. This idea is expressed by authors such as Toro [14] in 2007.

Regarding the sample size, the *COSMIN checklist* considers the following: the “small” size (<30 subjects) “poor”; the “moderate” sample size (30–49 participants) as “fair”; the samples between 50 and 99 participants as “good”; and “excellent” for those which consist of over 100 subjects [24]. In general, the reduced samples decrease the ability to extrapolate the results to the reference population [18]. It constitutes an item of four of the six metric properties assessed, which is decisive because the evaluation of each section on the *COSMIN checklist* is the lowest of the scores in their items [24]. Thus, a small sample size made seven of the articles [12, 26, 27, 29, 31, 33, 34] to be unable to obtain a higher score than “poor” in *Internal Consistency*, *Reliability*, *Criterion Validity*, and *Responsiveness*. Although the rest of the studies featured samples with more than 30 subjects, only two of them reached 50 participants [25, 36] which determined the results of *COSMIN checklist*.

The articles that registered the highest scores on the *COSMIN checklist* were the ones published by Dickens and Smith [28] and Chamorro-Moriana et al. [37] on the VGAS and the CHAGS scales, respectively. Only these two studies reached two scores of “fair.” This result partially coincides with that obtained in the *QUADAS-2* scale, in which studies by Dickens and Smith [28], Toro et al. [14], and Duque-Orozco et al. [15], on the SGT and EVGS levels, recorded the highest scores. They follow the articles published by Chamorro-Moriana et al. [37], Daly et al. [26], Mackey et al. [27], Macri et al. [17], and Leddy el al. [36]. The four studies that showed the highest scores were, in general, recent ones [14, 15, 28, 37]. All were published after 2000, and two of them were from 2016 [15, 37]. There were two articles subsequent to 2010 [35, 36]. Despite the low scores they obtained, the one published by Leddy et al. [36] stood out for its wide sample, composed of 80 patients, which is consistent with the increasing research requirements currently. According to the results, it can be considered that the observational gait assessment scale that has a higher methodological quality is VGAS [28], followed by CHAGS [37], SGT [14], and EVGS [29]. Three of these tools, VGAS [28], SGT [14], and EVGS [29], belong to neurology and, besides, are dedicated to assessing the gait of children with cerebral palsy (CP).

Due to the heterogeneity of articles, it was not appropriated to perform a meta-analysis thereof. That is, the spectrum of studies that were part of this project had differences in their characteristics, such as the target population, the sample size, or the metric properties.

Observational gait assessment scales are feasible tools for clinical practice. For this reason, many studies have recently been developed on their design, validation, and clinical applications. The existence of a wide variety of information makes its accessibility difficult for clinical and research areas. Thus, this systematic review is presented as a necessary compilation of gait assessment scales. It contains the study of the characteristics and the methodological quality of the scales validated to date. This operational document will therefore allow professionals to know the validated scales, to compare the tools available and to identify the more appropriate scale for each patient. It will be possible due to the analysis undertaken of each scale that includes the description of the tools: indications, language, operational instructions, items, and so on. Therefore, this study will allow the development of more precise and objective evaluations of gait. Consequently, it will improve the quality of the interventions, based on scientific evidence, optimize treatment times, and avoid relapses of the injury.

## 5. Limitations

The limitation of this paper was related to the selection criteria. They ensured that all included scales were validated. However, there exist traditional and well-known scales, such as *Observational Kinematic Gait Analysis*, which have not been included in this study since they are not validated. Therefore, we propose prospectively the development of validation studies on those scales that have been frequently employed in the clinical practice.

## 6. Conclusions

A compilation of gait assessment scales with no time limitation was conducted. Functional gait evaluation scales were systematically reviewed to identify the validated tools and analyze their characteristics and methodological quality. This operational document, available for clinical and research professionals, will lead to more precise and objective evaluations of gait. Thus, it will improve the quality of interventions in gait reeducation, based on scientific evidence, optimize treatment times, and avoid relapses.

Most validated scales were applied in neurological signs. The most approached topics in validated scales were orthopedic aids, phases of the gait cycle, and kinematics of the leg and trunk. All studies evaluated criterion validity, and fifteen of them analyzed intrarater or interrater reliability. The scale that demonstrated a higher methodological quality was VGAS, followed by CHAGS, SGT, and EVGS.

## Figures and Tables

**Table 1:** Search terms put into groups by mean.

Terms and strategies	Identifier
Gait OR walking OR locomotion OR ^*∗*^ambulation	1

Scale OR score OR questionnaire OR test OR criter^*∗*^ OR assess^*∗*^ OR analysis OR examination OR measure^*∗*^ OR outcome	2

“Gait scale” OR “gait score” OR “gait questionnaire” OR “walking scale” OR “walking score” OR “walking questionnaire” OR “locomotion scale” OR “locomotion score” OR “locomotion questionnaire” OR “gait test” OR “walking test” OR “locomotion test”	3

Observational OR visual	4

Valid ^*∗*^ OR reliability^a^	5

Criter^*∗*^: criteria, criterion; assess^*∗*^: assess, assessment; measure^*∗*^: measure, measurement; valid^*∗*^: valid, validation, validity; ^*∗*^ambulation: ambulation, deambulation. ^a^For PubMed database, these terms were substituted by the selection of *Validation studies* filter.

**Table 2:** Characteristics of the included scales and their validation.

Scale, author, year	Country of origin	Indications	Operating instructions and recommendations	Study sample	Metric properties
RVGA, Lord et al., 1998 [25]	United Kingdom	Neurological deficits	RVGA is composed of 20 items: 2 observations of the arms during the stance and swing phase and 18 observations of the trunk and legs (11 in the stand phase and 7 in the swing phase of the gait cycle). Items are scored from 0 to 3 points. The maximal punctuation of the scale refers to a much altered gait.	65 subjects (20 with multiple sclerosis)	Content V, criterion V, interrater R, responsiveness

GAIT, Daly et al., 2009 [26]	United States	Stroke	GAIT is made up of 31 items divided into 3 sections, which correspond to 3 phases of the gait cycle. Items have 3 possible scores: 0-1, 0–2, and 0–3 points. The maximum punctuation is 64 points that indicates a maximal deficit of the patient gait pattern. The estimated time by authors to use this assessment tool is 20 minutes.	29 subjects with stroke	Content V, criterion V, interrater R, intrarater R, responsiveness

SGT, Toro et al., 2007 [14]	United Kingdom	CP	SGT is used to describe the position of the trunk, hip, knee, and ankle of children with cerebral palsy during the 6 events of the gait cycle. The scale is composed of 18 evaluations: hip, knee, ankle, and trunk (normal, backwards, forwards). Items are scored from –2 to 2 points. The final amount reflects the gait pathology of the subject.	10 children with CP	Criterion V

OGS, Mackey et al., 2003 [27]	Australia	CP	OGS is composed of 8 items (scored from –1 to 3 points): 6 that evaluate movements and articular positions during gait cycle and 2 that analyze the need to employ assisted gait devices and the clinical evolution of the subject. The maximum punctuation (22 for each lower member) indicates a correct gait.	18 children with spastic CP	Criterion V, intrarater R, interrater R

GABS, Thomas et al., 2004 [4]	United States	Parkinson's disease	GABS evaluates gait, freeze of gait, gait cycle, balance, and posture. It consists of two parts: historical information and 14 parameters, evaluated in three possible ranges (0-1, 0–2, and 0–4). The first section is composed of questions relating to the basic activities of daily life, falls, and freeze of gait. The second section is divided into timed tasks (items 18–25) and nontimed tasks (items 26–28).	35 subjects with Parkinson's disease	Criterion V, intrarater R

VGAS, Dickens and Smith, 2006 [28]	United Kingdom	CP	VGAS is composed of 7 items, assessed in a range of 3 points (1 to 3) or five points (1 to 5). This scale analyzes the hip, knee, ankle, and foot position in the sagittal plane during the gait cycle events.	31 children with spastic hemiplegia	Criterion V, intrarater R, interrater R
Brown et al., 2008 [12]				4 children with spastic CP	Criterion V, intrarater R, interrater R

EVGS, Read et al., 2003 [29]	United Kingdom	CP	EVGS evaluates the position of the body segments in the sagittal, coronal, and transverse planes in a three-point scale (normal, moderate, and severe deviations). This scale is composed of 17 items scored from 0 to 2 points. The maximal alteration of gait is indicated by the result of 34 points. Zero points represent the absence of pathology.	4 children with CP and one normal control	Criterion V, intrarater R, interrater R, responsiveness
Duque-Orozco et al., 2016 [15]				30 children with spastic CP	Intrarater R, interrater R, criterion V

BAWI, Clarke and Eccleston, 2009 [30]	United Kingdom	Chronic pain	BAWI contains 11 items that assess the degree of variation of symmetry, responsiveness, and ability to follow test instructions. Items are evaluated in a range of 3 points (0–2). For symmetry, 0 indicated a symmetrical movement and 2, a bilaterally altered symmetry. Besides, BAWI considers aspects such as turning, movements of head and neck, and the use of aids during gait.	49 subjects with chronic pain	Internal consistency, criterion V, intrarater R, interrater R

HGAF, Hughes and Bell, 1994 [31]	United Kingdom	Hemiplegia	HGAF contains 18 items that evaluate the general characteristics of gait with a video recording, which gives an overall view of gait, swing phase, and stance phase. The various possible scores range from normal to definite abnormality.	6 subjects with hemiplegia	Criterion V, intrarater R, interrater R

OGA, Williams et al., 2009 [32]	Australia	Traumatic brain injury	OGA is composed of 20 items that evaluate spatiotemporal, kinematic, and kinetic aspects of gait. Items have 3 possible scores. All items are assessed as normal, increased, or decreased.	30 subjects with traumatic brain injury	Criterion V

SGS, Macri et al., 2002 [17]	Brazil	Fractures of the tibial shaft	SGS was designed to predict the healing of the tibial fractures. This scale consists of the classification of patient gait in a graduation (1 to 4): the first grade represents the extreme difficulty while grade 4 represents normal gait.	33 patients with a fracture of the tibial shaft	Criterion V, interrater R

SCI-FAI, Field-Fote et al., 2001 [33]	United States	Spinal cord injury	SCI-FAI is composed of 3 components. The first one evaluates gait parameters (e.g., step length and step rhythm). The second component analyzes the use of assisted devices. The third component assesses the distance and the time that the patient usually walks. The full scale contains 9 items evaluated in different ranges of punctuation. SCI-FAI includes a functional test, the 2-*minute walk test*. The result of 0 points represents the maximal alteration of gait.	22 subjects with spinal cord injury	Criterion V, intrarater R, interrater R, responsiveness

FGA, Wrisley et al., 2004 [34]	United States	Vestibular disorders Older adults Parkinson's disease	FGA is made up of 10 instructed tasks for the patient, as gait with horizontal head turns or gait and pivotal turn. As final punctuation of the tasks, which are evaluated from 0 to 3 points, 0 indicates a severe gait alteration and 3 corresponds to the development of a normal gait.	6 subjects with vestibular disorders	Internal consistency, structural V, criterion V, intrarater R, interrater R
Wrisley and Kumar, 2010 [35]				35 older adults	Content V, criterion V
Leddy et al., 2011 [36]				80 subjects with Parkinson's disease	Criterion V, intrarater R, interrater R

CHAGS, Chamorro-Moriana et al., 2016 [37]	Spain	Sprained ankle	CHAGS is an assessment scale of assisted gait with one or two forearm crutches. It is comprised of 10 items evaluated in a range of 5 points (0–4). The interpretation of the scale has to be performed item by item and not globally. Thus, a result of 4 points in each item indicates a correct gait, a punctuation of 3 is considered acceptable, and a result ≤2 corresponds to a nonacceptable gait.	30 subjects with sprained ankle	Internal consistency, content V, criterion V, intrarater R, interrater R

RVGA: Rivermead Visual Gait Assessment; GAIT: Gait Assessment and Intervention Tool; SGT: Salford Gait Tool; OGS: Observational Gait Scale; GABS: Gait and Balance Scale; VGAS: Visual Gait Assessment Scale; EVGS: Edinburgh Visual Gait Score; BAWI: Bath Assessment of Walking Inventory; HGAF: Hemiplegic Gait Analysis Form; OGA: Observational Gait Analysis; SGS: Standardised Gait Score; SCI-FAI: Spinal Cord Injury Functional Ambulation Inventory; FGA: Functional Gait Assessment; CP: cerebral palsy; V: validity; R: reliability.

**Table 3:** Data approached by items of scales.

Scales	Kinematics			Kinetics	Spatial parameters (step length, step width)	Temporal parameters (velocity, cadence)	Gait cycle phases	Fluency	Arm swing	Facing forward	Center of gravity (base of support displacement)	Gait optimization parameters (jumps/displacements)	Falls	Balance	Gait with obstacles	Orthopedic aids	Functional Tests	Psychological aspects (confidence etc.)	Level of care
	Leg	Arm	Trunk																
1. RVGA (Lord et al. [25])	✓	✓	✓				✓									✓			
2. GAIT (Daly et al. [26])	✓	✓	✓				✓		✓		✓	✓				✓			
3. SGT (Toro et al. [14])	✓		✓				✓												
4. OGS (Mackey et al. [27])	✓				✓		✓									✓			
5. GABS (Thomas et al. [4])			✓		✓	✓	✓	✓	✓			✓	✓	✓	✓	✓	✓	✓	✓
6. VGAS (Dickens and Smith [28], Brown et al. [12])	✓						✓												
7. EVGS (Read et al. [29], Duque-Orozco et al. [15])	✓		✓				✓												
8. BAWI (Clarke and Eccleston [30])			✓		✓				✓			✓			✓	✓			
9. HGAF (Hughes and Bell [31])	✓	✓	✓		✓	✓	✓				✓								
10. OGA (Williams et al. [32])	✓		✓	✓	✓	✓					✓								
11. SGS (Macri et al. [17])						✓										✓			
12. SCI-FAI (Field-Fote et al. [33])					✓	✓										✓	✓		
13. FGA (Wrisley et al. [34])						✓		✓				✓		✓	✓	✓			
14. CHAGS (Chamorro-Moriana et al. [37])					✓	✓		✓	✓	✓	✓					✓			

RVGA: Rivermead Visual Gait Assessment; GAIT: Gait Assessment and Intervention Tool; SGT: Salford Gait Tool; OGS: Observational Gait Scale; GABS: Gait and Balance Scale; VGAS: Visual Gait Assessment Scale; EVGS: Edinburgh Visual Gait Score; BAWI: Bath Assessment of Walking Inventory; HGAF: Hemiplegic Gait Analysis Form; OGA: Observational Gait Analysis; SGS: Standardised Gait Score; SCI-FAI: Spinal Cord Injury Functional Ambulation Inventory; FGA: Functional Gait Assessment.

**Table 4:** Assessment of the methodological quality with QUADAS-2.

Study	Risk of bias				Applicability		
	Patient selection	Index test	Reference standard	Flow and timing	Patient selection	Index test	Reference standard
Lord et al. [25]	?	?	?	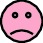	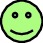	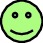	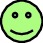
Daly et al. [26]	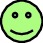	?	?	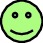	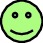	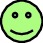	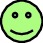
Toro et al. [14]	?	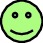	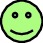	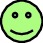	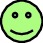	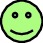	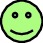
Mackey et al. [27]	?	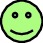	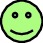	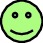	?	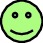	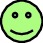
Thomas et al. [4]	?	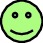	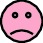	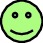	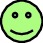	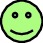	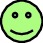
Dickens and Smith [28]	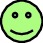	?	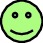	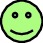	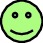	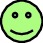	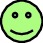
Brown et al 2008 [12]	?	?	?	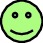	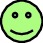	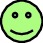	?
Read et al. [29]	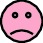	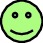	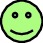	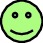	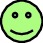	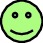	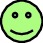
Duque-Orozco et al. [15]	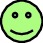	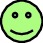	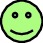	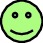	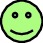	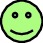	?
Clarke and Eccleston [30]	?	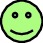	?	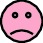	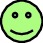	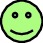	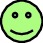
Hughes and Bell [31]	?	?	?	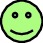	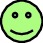	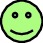	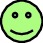
Williams et al. [32]	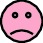	?	?	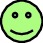	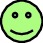	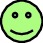	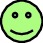
Macri et al. [17]	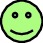	?	?	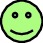	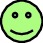	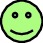	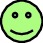
Field-Fote et al. [33]	?	?	?	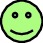	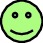	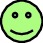	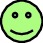
Wrisley et al. [34]	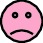	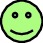	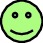	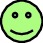	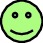	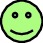	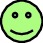
Wrisley and Kumar [35]	?	?	?	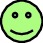	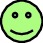	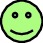	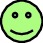
Leddy et al. [36]	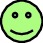	?	?	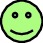	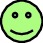	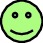	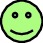
Chamorro-Moriana et al. [37]	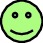	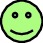	?	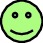	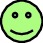	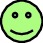	?

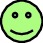
: low risk of bias or low concerns regarding applicability; 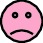: high risk of bias or high concerns regarding applicability; ?: unclear risk of bias or unclear concerns regarding applicability.

**Table 5:** Assessment of methodological quality with *COSMIN checklist*.

Study	Internal consistency^a^	Reliability^a^	Content validity	Structural validity^a^	Criterion validity^a^	Responsiveness^a^
RVGA Lord et al. [25]	–	+	+	–	+	+
GAIT Daly et al. [26]	–	+	+	–	+	+
SGT Toro et al. [14]	–	–	–	–	++	–
OGS Mackey et al. [27]	–	+	–	–	+	–
GABS Thomas et al. [4]	–	+	–	–	+	–
VGAS Dickens and Smith [28]	–	++	–	–	++	–
VGAS Brown et al. [12]	–	+	–	–	+	–
EVGS Read et al. [29]	–	+	–	–	+	+
EVGS Duque-Orozco et al. [15]	–	+	–	–	+	–
BAWI Clarke and Eccleston [30]	+	+	–	–	++	–
HGAF Hughes and Bell [31]	–	+	–	–	+	–
OGA Williams et al. [32]	–	–	–	–	++	–
SGS Macri et al. [17]	–	++	–	–	+	–
SCI-FAI Field-Fote et al. [33]	–	+	–	–	+	+
FGA Leddy et al. [36]	–	+	–	–	++	–
FGA Wrisley et al. [34]	+	+	–	++	+	–
FGA Wrisley and Kumar [35]	–	–	+	–	++	–
CHAGS Chamorro-Moriana et al. [37]	+	++	+	–	++	–

RVGA: Rivermead Visual Gait Assessment; GAIT: Gait Assessment and Intervention Tool; SGT: Salford Gait Tool; OGS: Observational Gait Scale; GABS: Gait and Balance Scale; VGAS: Visual Gait Assessment Scale; EVGS: Edinburgh Visual Gait Score; BAWI: Bath Assessment of Walking Inventory; VAHG: Visual Assessment of Hemiplegic Gait; OGA: Observational Gait Analysis; SGS: Standardised Gait Score; SCI-FAI: Spinal Cord Injury Functional Ambulation Inventory; FGA: Functional Gait Assessment; +: poor; ++: fair; +++: good; ++++: excellent. ^a^Metric properties that include sample size assessment.

## Supplementary Materials

10.1371/journal.pmed100009 Supplementary Materials Search strategies: it is a table which contains the complete search strategies applied to each electronic database, including the filter selected in PubMed database, as well as a simplification of each strategy. PRISMA statement checklist: it contains the checklist published by Moher et al. (Moher D, Liberati A, Tetzlaff J, Altman DG, The PRISMA Group (2009). Preferred Reporting Items for Systematic Reviews and Meta-Analyses: The PRISMA Statement. PLoS Med 6(7): e1000097. doi: 10.1371/journal.pmed100009), which addresses several conceptual and practical advances in the science of systematic reviews.
